# A vaccine revertant of highly pathogenic porcine reproductive and respiratory syndrome virus: re-emergence after lurking for 12 years

**DOI:** 10.1128/spectrum.00728-25

**Published:** 2025-05-22

**Authors:** Weixin Wu, Lin Lin, Zian Ye, Ruoning Hou, Qiongqiong Zhou, Lei Zhou, Hanchun Yang

**Affiliations:** 1 National Key Laboratory of Veterinary Public Health Safety, College of Veterinary Medicine, China Agricultural University 630101 , Beijing, China; 2 Key Laboratory of Animal Epidemiology of Ministry of Agriculture and Rural Affairs, College of Veterinary Medicine, China Agricultural University 630101 , Beijing, China; 3 College of Veterinary Medicine, Northeast Agricultural University 12430, Harbin, Heilongjiang, China; University of Prince Edward Island, Charlottestown, Prince Edward Island, Canada

**Keywords:** porcine reproductive and respiratory syndrome virus, vaccine, virulence reversion, outbreak

## Abstract

Porcine reproductive and respiratory syndrome virus (PRRSV) is an economically important pathogen for the global pork industry. Modified live virus (MLV) vaccines are widely used for PRRSV prevention and control. However, MLV vaccines can still infect hosts and replicate in the target cells, increasing the risk of virus recombination and reversion to virulence. Recently, a highly pathogenic PRRSV strain appeared in December 2024, causing severe outbreaks on a PRRSV-negative pig farm in Yunnan, China. The isolated strain exhibited 99.4%–99.5% nucleotide similarity to the historical JXA1-R vaccine-derived revertant isolated in 2012, despite the farm never using JXA1-R vaccines. Ten unique amino acid mutations confirmed that this strain was a revertant derived from JXA1-R vaccines, which reverted to cause an outbreak in 2012 and vanished for 12 years.

**IMPORTANCE:**

Currently, there is a relative consensus that porcine reproductive and respiratory syndrome modified live virus (PRRS MLV) has the highest protective efficacy against the genetically homologous virus, compared with commercially available killed vaccines or other kinds of vaccines under development. However, risks of virulence reversion remain in such live vaccines. This case reveals the unpredictable risks of live-attenuated vaccines. The findings underscore the inherent flaws in highly pathogenic PRRSV MLV vaccines, emphasizing the need for safer vaccine designs. The re-emergence of that revertant draws our attention to its source, and the similar mutation site compared with 12 years ago could indicate key nucleotide sites for the reversion of virulence of this strain.

## OBSERVATION

Porcine reproductive and respiratory syndrome virus (PRRSV) is characterized by its high contagiousness and genetic variation, posing significant threats to pig health (1). In 1996, the first PRRSV strain (CH-1a) of the Chinese mainland was isolated from an aborted fetus on a pig farm. Subsequently, atypical PRRS characterized by high fever and high mortality, caused by highly pathogenic PRRSV (HP-PRRSV), broke out in China in 2006, then quickly spread to over 10 provinces (autonomous cities or regions) and affected over 2,000,000 pigs with about 400,000 fatal cases (2).

After that, several HP-PRRSV-derived MLVs, including JXA1-R, were commercialized and widely used (3). Unfortunately, extensive utilization of MLVs seriously increases the risk of reversion to virulence. In 2012, three isolates exhibiting high nucleotide similarity and the presence of specific nucleotide mutation sites similar to those of the HP-PRRSV-derived vaccine strain JXA1-R caused severe outbreaks and were isolated from field samples (4). The utilization of HP-PRRSV-derived MLV has undergone a gradual decline since that time. During 2013 and 2014, the NADC30-like strain was introduced and isolated in Mainland China (5, 6), and it has gradually become the dominant strain, or the dominant recombination isolate donor strain with HP-PRRSV MLV in recent years. Despite some JXA1-R-like recombinants being isolated from field samples (7), no severe outbreak or “abortion storm” related to these viruses has been documented for 12 years.

**Fig 1:**
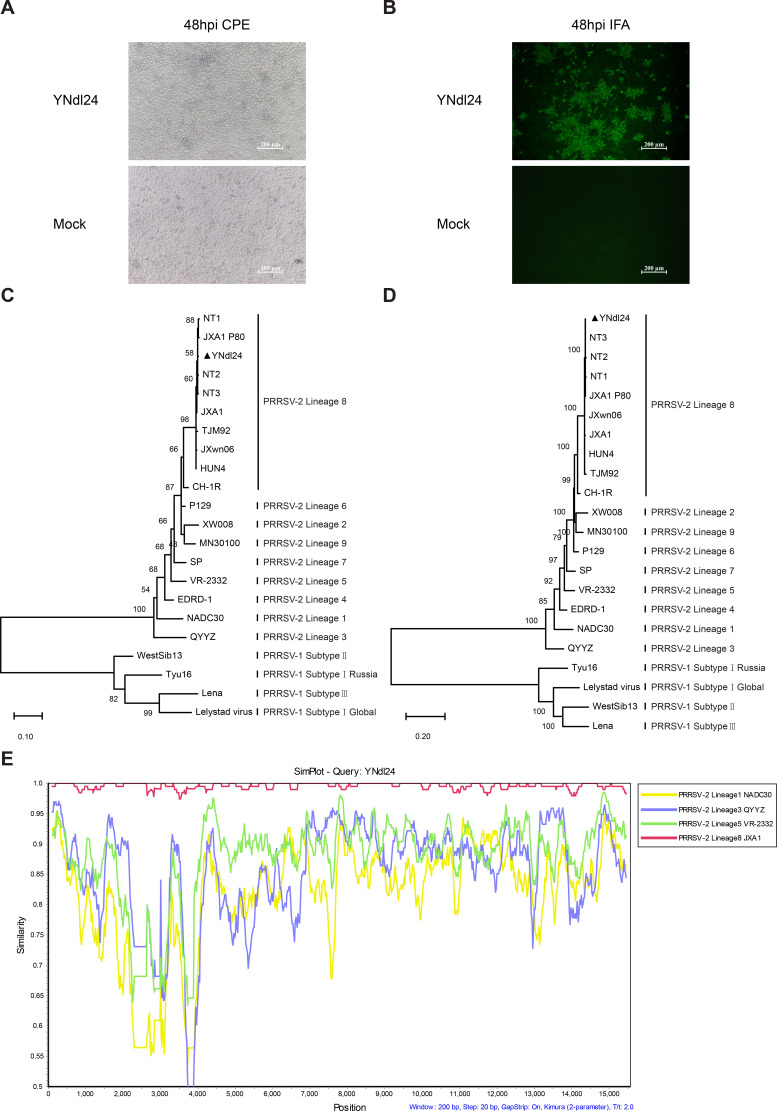
Isolation and characterization of YNdl24. (**A**) CPE of YNdl24 on MARC-145 cells. (**B**) IFA identification of YNdl24 on MARC-145 cells. (**C**) Phylogenetic tree based on PRRSV ORF5 genes was constructed by the maximum likelihood method in MEGA12 (version 12.0.4), with the Kimura 2-parameter model, and the bootstrap value was set to 500. (**D**) Phylogenetic tree based on PRRSV full-length genome was constructed by the identical parameter settings utilized in this analysis as described previously. (**E**) Recombination analysis was conducted by Simplot (version 3.5.1).

From December 2024 to January 2025, the abortion rate (including aborted fetuses, mummified fetuses, stillborn, and nonviable neonatal piglets) of sows’ herds suddenly increased, along with high fever in piglets, which was observed on a PRRSV-negative intensive breeding pig farm in Yunnan, a southwest province of China. Etiological investigations revealed positive results in sows and gilts, with stillbirth and abortion rates up to 88%, indicating a severe outbreak of PRRS. The causative virus was successfully isolated from the sera of aborted pigs by inoculating into MARC-145 cells (ATCC, CRL-12231), following a previously described procedure (8), and exhibited cytopathic effect (CPE) characterized by cell congregation, contraction, and eventually brushing off (Fig. 1A). The isolate was confirmed by IFA using anti-PRRSV N mAb (Fig. 1B) and designated as YNdl24.

**Fig 2:**
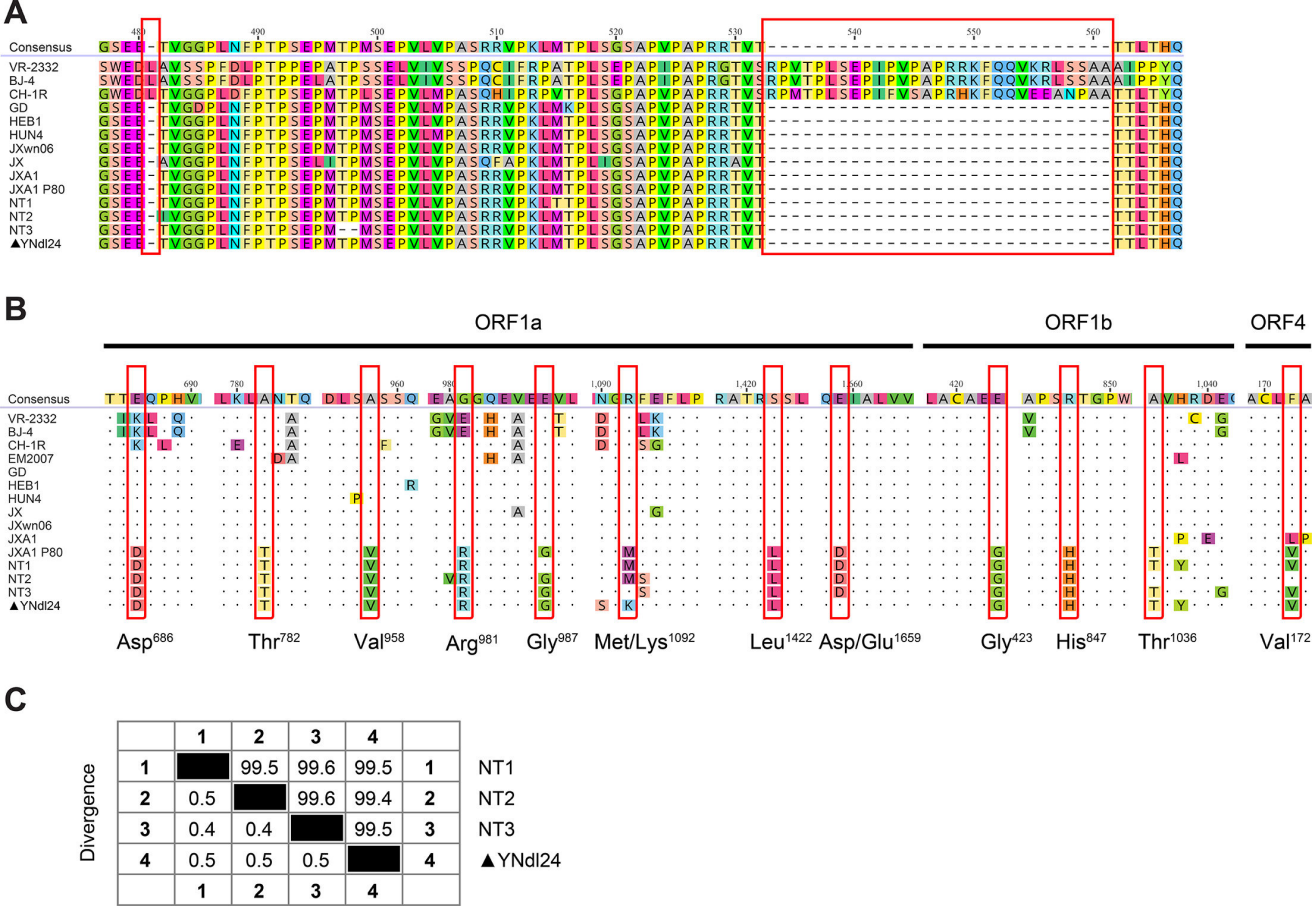
Sequence analysis of YNdl24. (**A**) Analysis of the NSP2 deletion pattern of YNdl24 was conducted by Geneious Prime (version 2024.0.5). (**B**) Analysis and comparisons of partial ORF1a, ORF1b, and ORF4 deduced amino acid sequences from the PRRSV strains were conducted by Geneious Prime (version 2024.0.5) using PRRSV VR-2332 as a reference for amino acid positions. Unique and identical amino acids among YNdl24 and JXA1-R derived strains are marked with red boxes. (**C**) Sequence distance of YNdl24 with NT1, NT2, or NT3 was calculated by Malign (version 11.1.0).

Subsequent passages of the virus in MARC-145 cells were performed, and the virus titer of passage 3 was determined to be 10^7^ TCID_50_/mL by using the Reed-Muench method in MARC-145 cells. The genomic RNA of viruses from the second passage was extracted and amplified using reverse transcription PCR with 14 pairs of overlapping primers (Table S1). The full-length genome was sequenced and submitted to GenBank with the number PQ863973. A blast search in the publicly accessible database of GenBank revealed a high degree of nucleotide homology (exceeding 99.4%) with different passages of the JXA1 strain, which have been produced during attenuation for vaccines. Phylogenetic analysis of ORF5 (Fig. 1C) and the entire genome (Fig. 1D) further demonstrated that the isolate was classified as *Betaarterivirus americense* (also known as the North American strain, or PRRSV-2) lineage 8 virus and was in the same branch as JXA1 P80. The absence of recombination signals within PRRSV-2 lineages (Fig. 1E) indicates that the isolate was an unreconstituted HP-PRRSV strain, exhibiting a consistent Nsp2 discontinuous deletion pattern (Fig. 2A). It is noteworthy that the farm never utilized the JXA1-R vaccine. Instead, it exclusively introduced PRRSV naive gilts. Moreover, the implementation of JXA1-R vaccination would not directly result in pathogenesis. So, the severe outbreak in this case may be reminiscent of the NT1, NT2, and NT3, the early revertants of the JXA1-R vaccine, isolated in Jiangsu province in 2012 (4). A startling alignment indicates that YNdl24 shares 10/12 common amino acid mutations that are exclusively present in JXA1-R-derived strains (Fig. 2B), and the nucleotide similarity ranged from 99.4% to 99.5% with NT-1, NT2, or NT3 (Fig. 2C). The clinical signs exhibited by the piglets included high fever (≥40.5°C), respiratory signs of distress, and anorexia, which are analogous to the observations made in animal trials of NT1, NT2, or NT3 (4).

Given the high mutagenesis rate of PRRSV evolution, it is not feasible to maintain such a high degree of homology during repeated circulation in the field for 12 years before resurfacing and causing a severe outbreak. If the vaccine strain recently reverted to a virulent strain, what selective pressure could drive its evolution to resemble that of 12 years ago? The similar mutation site throughout 12 years suggests that the crucial sites associated with pathogenicity remain worthy of further study. Moreover, the potential for this strain to spread extensively and re-emerge as the predominant strain is another matter of concern. Anyhow, our previous study has presented direct evidence for the reversion to virulence of HP-PRRSV MLV after serial passaging in pigs (9), and the virulence reversion strains isolated from the field have exposed the fatal flaws inherent in HP-PRRSV-derived vaccines.

## SUPPLEMENTAL MATERIAL

Table S1 spectrum.00728-25-s0001.xlsx Primers used for PRRSV sequencing in this study.

ASM does not own the copyrights to Supplemental Material that may be linked to, or accessed through, an article. The authors have granted ASM a non-exclusive, world-wide license to publish the Supplemental Material files. Please contact the corresponding author directly for reuse.
